# The association between neutrophil lymphocyte ratio and perihematomal edema in cerebral hemorrhage: a multicenter retrospective study

**DOI:** 10.3389/fneur.2025.1575446

**Published:** 2025-07-04

**Authors:** Yanwei Liu, Qi Liu, Jun Wei, Shiqiang Yang

**Affiliations:** ^1^Department of Neurology, The First People’s Hospital of Yibin, Yibin, Sichuan, China; ^2^Department of Neurosurgery, Second Chinese Medicine Hospital of Sichuan Province, Chengdu, Sichuan, China; ^3^Department of Neurosurgery, The First People’s Hospital of Yibin, Yibin, Sichuan, China; ^4^Department of Neurosurgery, West China Hospital of Sichuan University, Chengdu, Sichuan, China

**Keywords:** neutrophil lymphocyte ratio, perihematomal edema, hypertensive cerebral hemorrhage, restricted cubic spline, logistic regression analysis

## Abstract

**Background:**

Perihematomal edema (PHE) after a brain hemorrhage is an increase in the water content of the brain tissue surrounding the haematoma, which can be observed and measured on imaging. PHE is one of the major secondary brain injuries after a brain hemorrhage and is strongly associated with poor patient prognosis. The relationship between the neutrophil-lymphocyte ratio (NLR) and cerebral oedema after cerebral hemorrhage remains unclear.

**Methods:**

Data for this study were obtained from a registry database of hospital admissions at two medical institutions covering the population of southwest China. The researchers compared outcomes, including demographics, medical history and lesion characteristics, for all included cases. The primary exposure factor for this study was NLR on admission (NLR1), while NLR measured between 3 and 5 days of treatment (NLR2) was used as a secondary exposure factor for comparison. The study outcome was the degree of PHE after 5–7 days of standardized treatment. The association between NLR and PHE was examined using Restricted cubic spline (RCS) and Logistic regression modeling, and absolute rate differences and risk ratios with 95% confidence intervals were calculated.

**Results:**

A total of 143 patients with confirmed hypertensive cerebral hemorrhage were finally included. Their mean age was 52.8 ± 9.1 years and 53.1% were female. Restricted cubic spline analysis suggested a linear positive correlation between admission NLR and PHE. Logistic regression analysis adjusted for covariates showed that admission NLR was significantly associated with the risk of developing moderate to severe PHE (OR, 1.61 [95% CI, 1.03–2.5]; *p* = 0.035). In addition, NLR was divided into higher and lower groups according to the median and then analyzed by logistic model regression with multiple covariate adjustment. The results showed that a higher NLR was significantly associated with a higher risk of moderate to severe PHE compared to the lower group (OR, 2.47 [95% CI, 1.59–3.92]; *p* = 0.021). These results remained stable in subsequent subgroup and sensitivity analyses.

**Conclusion:**

Admission NLR was significantly and linearly positively correlated with PHE. In clinical practice, admission NLR can be used as a predictor of potentially moderate to severe PHE. However, further research is needed to explore and explain this due to potential residual confounders.

## Introduction

According to the latest Global Burden of Disease (GBD) study, the global death rate from intracerebral hemorrhage (ICH) is expected to increase by 27% by 2050 compared with 2019 (1). This increase is projected to be more pronounced in regions with lower socioeconomic levels, highlighting the significant impact of ICH on global health (2). Effective strategies to manage and prevent ICH, particularly in these vulnerable populations, are therefore of utmost importance. According to the latest guidelines and research, the management of brain hemorrhage includes tight control of hypertension and clinical symptomatic management after bleeding (3). For example, the 2015 American Heart Association/American Stroke Association guidelines provide best-evidence-based recommendations for the early management of cerebral hemorrhage, including prehospital care, emergency evaluation and treatment, and inpatient management (4). Cerebral oedema is a pathological condition following brain injury characterized by an increase in brain tissue volume due to fluid accumulation. Cerebral oedema is an important complication of several neurological disorders, including stroke, traumatic brain injury and brain tumors. It is associated with increased intracranial pressure, neurological deficits and a poor clinical prognosis (5). Understanding the pathophysiological mechanisms of cerebral oedema is essential for the development of effective therapeutic strategies. Recent areas of research and future directions include the study of mechanisms such as inflammatory pathways, the application of neuroimaging techniques and personalized treatment (6, 7). Developments in these areas will help to improve the outcome and prognosis of hypertensive cerebral hemorrhage.

Neutrophils are key players in the inflammatory response and can lead to disruption of the blood–brain barrier (BBB) and exacerbation of cerebral oedema (8). In contrast, lymphocytes are involved in regulating the immune response and are associated with resolution of inflammation (9). The neutrophil-lymphocyte ratio (NLR) is a readily measurable biomarker of systemic inflammation that plays an important role in the pathological process of cardiovascular disease, and an elevated NLR is associated with poor prognosis in tumor patients, including tumor aggressiveness, metastasis and survival (10). In addition, NLR has been significantly correlated with the severity and acute exacerbation of chronic obstructive pulmonary disease (COPD), the diagnosis and prognosis of infectious diseases, and the activity and prognosis of autoimmune diseases (11–13). Therefore, NLR is increasingly recognized as a predictor of disease severity and prognosis in a variety of clinical settings. A study by Zhu et al. (14) showed that patients with acute ischaemic stroke and high NLR levels had more severe brain oedema on imaging. Similarly, Siwicka-Gieroba et al. (15) found that NLR was significantly correlated with the degree of brain oedema in patients with traumatic brain injury. Recent studies have shown that NLR can be used as a predictor of prognosis in patients with brain injury and that increased NLR may be associated with an enhanced inflammatory response after brain injury, which plays an important role in the pathological process of brain injury (16). In patients with acute ischaemic stroke with large vessel occlusion, NLR is an independent predictor of malignant cerebral oedema after successful revascularization (17).

Despite these preliminary findings, the exact relationship between NLR and cerebral oedema, especially in the context of different neurological disorders, remains to be fully elucidated. In addition, the underlying mechanisms by which NLR influences the development and regression of cerebral oedema are poorly understood. This knowledge gap hinders the clinical application of NLR as a prognostic tool and therapeutic target. The aim of this study was to investigate the relationship between NLR as a continuous variable and perihematomal oedema after cerebral hemorrhage. We hypothesized that NLR is an important predictor of the severity of perihematoma oedema. We sought to gain a more nuanced understanding of the role of NLR at different time points in this complex pathological process.

## Methods

### Study design

This retrospective observational cohort study used data from the electronic case management system of Chinese public hospitals. The cohort was drawn from the Second Traditional Chinese Medicine Hospital of Sichuan Province and the First People’s Hospital of Yibin City in southwest China. Thus, this cohort is representative of the Chinese population of different income levels, ethnicities and religions in southwest China. The data used in our study were extracted from the electronic case management systems of these two large medical institutions. These records included demographic information, diagnostic and treatment details, and comprehensive imaging data.

The study protocol was approved by the Ethics Committee of the First People’s Hospital of Yibin City. Although our retrospective observational cohort study involved the collection and analysis of patient data, informed consent was not required due to the use of unidentified past medical records. The study was conducted in accordance with relevant ethical and privacy regulations, and all patient data were anonymized and treated with the utmost confidentiality to ensure compliance with ethical standards. In addition, the study protocol was officially registered with the China Clinical Trial Registry under the registration number ChiCTR2300070907, and this study was reported in accordance with the Enhanced Reporting of Observational Studies in Epidemiological Statements (18).

### Participants

**Figure 1:**
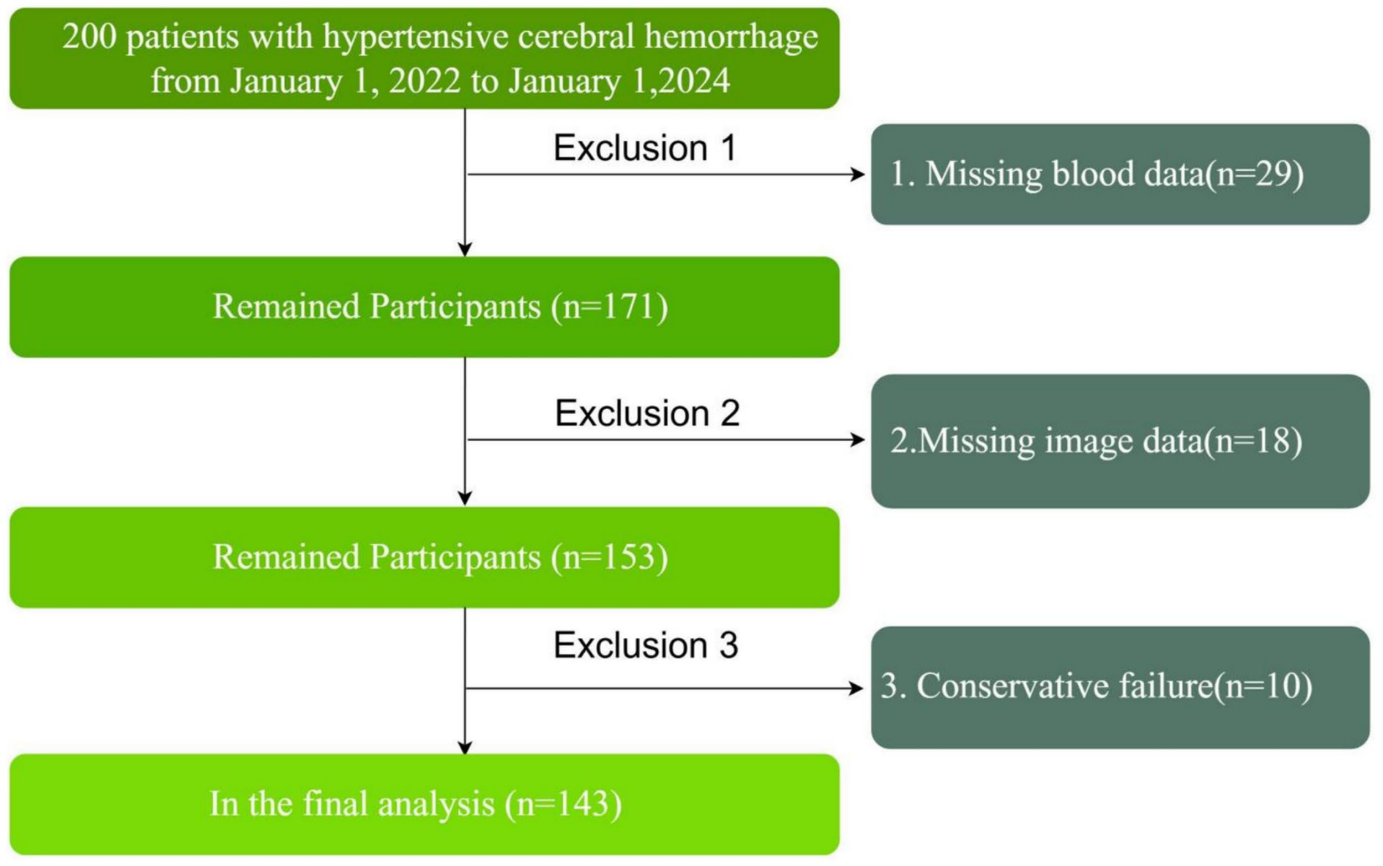
Study design and flowchart.

In this study, a retrospective cohort study was conducted to evaluate the relationship between NLR and PHE after hypertensive cerebral hemorrhage at different time points. The outcome event of the study was the degree of oedema at the time of worst cerebral oedema 5–7 days after treatment. To ensure a homogeneous patient cohort focused on those primarily managed with conservative therapy, we selected patients with moderate volumes of cerebral hemorrhage. Specifically, we included patients with supratentorial cerebellar hemorrhages of less than 30 mL, and infratentorial cerebellar hemorrhages of less than 10 mL. These thresholds were chosen based on clinical guidelines and previous research indicating that hematomas exceeding these volumes are more likely to necessitate surgery. By selecting patients with hematomas below these thresholds, we aimed to minimize the likelihood of surgical intervention and ensure that our cohort was representative of patients who would typically receive conservative treatment. This approach also enabled us to focus on a group of patients in whom the effects of conservative treatment on PHE could be evaluated more consistently. These volume thresholds define a ‘moderate volume’ group that is more likely to be managed conservatively, providing a more accurate assessment of the relationship between NLR and PHE in this clinical context. Once the database was established, relevant study indicators were extracted and data cleaning was performed. First, 29 cases with missing blood cell data during the study period were deleted. Next, 18 cases with missing imaging data during the study period were deleted. Finally, 10 cases with changes in treatment protocol due to changes in condition, such as rebleeding during conservative treatment, were excluded (Figure 1).

### Data collection

Baseline characteristics of the study population were extracted from the eCase system, including demographic factors, diagnostic status and treatment information. Demographic factors included sex, age and BMI at the time of treatment. Diagnostic conditions included admission GCS score, NIHSS score, location of haematoma, whether the haematoma was in the ventricle, and volume of hemorrhage. Treatment information included data on cerebral haematoma volume, cerebral oedema volume and oedema index after treatment. Missing data handling: To address this issue, we implemented a rigorous data cleaning process. Specifically, we excluded cases with missing blood cell (*n* = 29) or imaging (*n* = 18) data during the study period. We also excluded 10 cases where the treatment protocol was changed due to rebleeding during conservative treatment. These exclusions ensured that our analysis was based on complete and reliable data. For any remaining variables with missing data, we used multiple imputation techniques to estimate the missing values and prevent bias in our analysis due to incomplete data. Consistency measures were employed in data extraction. To ensure consistency and accuracy, we used a standardized protocol for data extraction. Two independent researchers extracted data from the electronic case management system and cross-checked it for accuracy. Any discrepancies were resolved through discussion and reference to the original medical records. Additionally, we conducted a pilot study on a subset of the data to refine our extraction methods and ensure consistency. This approach minimized the risk of errors and guaranteed that the data used in our analysis were accurate and consistent.

### Exposure and outcome evaluation

The main exposure factor of interest in this study was the neutrophil-to-lymphocyte ratio (NLR), as it is a dynamic indicator. Therefore, neutrophil and lymphocyte counts were extracted from the initial blood indices obtained upon admission. The resulting NLR1 value represents the patient’s inflammatory response at the time of admission. As inflammatory states change dynamically during treatment, the NLR2 value from days 3–5 was used as a secondary exposure factor. To account for potential variability within this timeframe, the average NLR measurement was calculated from the values taken between days three and five. If multiple measurements were available within this period, the average was calculated to represent the overall inflammatory state during this critical period. This approach minimizes the impact of transient fluctuations and provides a more stable measure of NLR during treatment.

**Figure 2:**
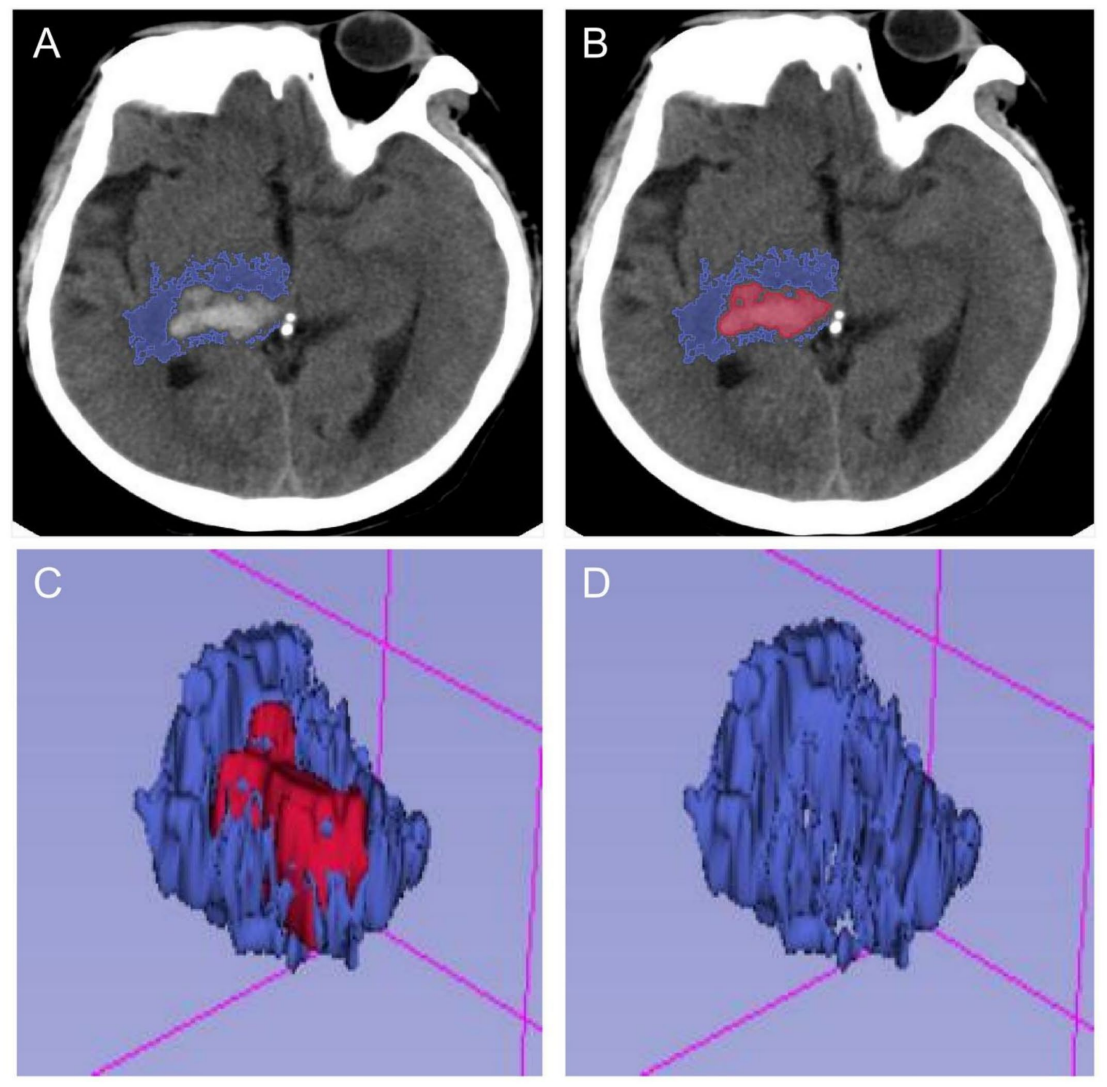
Schematic diagram of calculating PHE after processing by 3D-Slicer software. **(A,B)** The ranges of hematoma and edema were delineated in each section of head CT, respectively. **(C,D)** The volumes of hematoma and edema in the brain were calculated in the open-source 3D-Slicer software, and the edema index was calculated.

The primary outcome of this study was the extent of PHE at the peak of cerebral oedema. PHE is usually identified using computed tomography (CT) scans. On a CT scan, it appears as a hypodense area around the haematoma. In this study, the volumes of the haematoma and oedema were measured using open-source 3D Slicer software (Figure 2). The oedema index (EI) was then calculated by taking the ratio of the oedema volume at the peak to the haematoma volume at admission: EI = (oedema volume–haematoma volume)/haematoma volume. Based on previous clinical studies, patients were categorized as having mild oedema if their EI was less than 1.5. Those with an EI greater than 1.5 were defined as having moderate to severe oedema. This threshold has previously been used to differentiate between mild and more severe forms of PHE (9).

### Conservative treatment

In western China, the clinical promotion and popularity of minimally invasive techniques is low. Unwilling to accept the risks associated with surgical treatment, conservative therapy is therefore the preferred treatment option for neurologists, awake patients and their families. A combination of therapeutic approaches is currently used in accordance with guidelines for conservatively treated patients. This includes protocols such as routine blood pressure control, management of intracranial pressure to reduce dehydration, nutritional support to relieve symptoms, maintenance of water-electrolyte balance and implementation of anti-infective measures to reduce potential complications. And during the treatment period, head CT and other investigations were not regularly reviewed to assess the conditions of re-bleeding and cerebral oedema.

### Statistical analysis

All analyses were performed using the R 3.3.2 statistical package (http://www.R-project.org, The R Foundation, Shanghai, China) and Free Statistic V1.9 statistical software. Statistical analyses used in this study included the *χ*^2^ test or Fisher’s exact test for categorical variables, and Student’s *t*-test for continuous variables. To explore the potential association between NLR and perihematoma oedema, this study used restricted cubic spline to analyze the potential relationship between NLR and outcome. Univariate and multivariate logistic regression analysis and model analysis were also used. In addition, subgroup analyses were performed to verify the stability of the results.

The multivariate analysis focused on analyzing the relationship between NLR as the dependent variable and the study outcome (oedema index) under the influence of different covariates. Therefore, we developed four models for multivariate analysis. In model 1, we did not adjust for any covariates. In model 2 we adjusted for two covariates, age and sex. In model 3, we continued to adjust for the covariates of haematoma location, whether it infiltrated the ventricle, GCS and NIHSS based on model 2. In model 4, we continued to adjust for the covariates of BMI, history of hypertension, history of diabetes mellitus, and smoking and drinking status based on model 3.

## Results

### Patient numbers

Systematic collection of clinical data for this study began in February 2024. In China, the Chinese Guidelines for the Diagnosis and Treatment of Cerebral Haemorrhage (2019) was jointly developed by the Neurology Section of the Chinese Medical Association and the Cerebrovascular Disease Group of the Neurology Section of the Chinese Medical Association. It aims to reflect the latest standards in the diagnosis and treatment of acute cerebral hemorrhage and provide guidance to clinicians. The guideline suggests that surgical decompression may be considered for haematoma volumes greater than 30 mL above the supratentorial hemorrhage and greater than 10 mL below the infratentorial hemorrhage. Therefore, to more accurately calculate the observed outcomes of cerebral hemorrhage and peri-haematoma oedema, we selected patients with moderate volume cerebral hemorrhage who received standardized conservative management. We extracted inpatients diagnosed with hypertensive cerebral hemorrhage between 1 January 2022 and 1 January 2024 from the electronic case management systems of two large healthcare institutions. Then, 200 patients diagnosed with supratentorial cerebellar hemorrhage with a haematoma volume of less than 30 mL or infratentorial cerebellar hemorrhage with a haematoma volume of less than 10 mL were randomly selected. Once the database was established, relevant study indicators were extracted and data cleaning was performed. First, 29 cases with missing hematological data during the study period were deleted. Next, 18 cases with missing imaging data during the study period were removed. Finally, 10 cases with a change in treatment regimen due to a change in condition (rebleeding during conservative treatment) were removed. In the end, 143 patients were included in this study (Figure 1).

### Basic characteristics

Of a total of 143 individuals in this cohort, 67 (46.9%) were male with a mean age of (52.8 ± 9.1) years. The mean admission hemorrhage volume for all participants was 14.9 ± 4.8 ML, and at the peak of subsequent oedema the measured volume of perihematomal oedema was 22.3 ± 8.1 ML. In addition, 46.2% of all participants had a long history of cigarette smoking, 39.8% had a long history of alcohol consumption, 11.2% had comorbid diabetes mellitus, and 92.3% suffered from hypertensive disorders (further detailed results are shown in Table 1). Of the 200 randomly selected patients, 10 were excluded due to changes in their condition during conservative treatment and were treated with other modalities. In contrast, the remaining patients passed the peak oedema phase during conservative treatment and continued to be treated with neurorehabilitation.

**Table 1:** Description of demographic and clinical characteristics.

Variables	Degree of perihematoma edema (PHE)			*p*
	Total (*N* = 143)	Mildly (EI < 1.5, *N* = 94)	Moderate to severe (EI ≥ 1.5, *N* = 49)	
Age (year), Mean± SD	52.8 ± 9.1	53.3 ± 9.1	52.2 ± 9.3	0.489
Gender, *n* (%)				0.075
Male	67 (46.9)	39 (41.5)	28 (57.1)	
Female	76 (53.1)	55 (58.5)	21 (42.9)	
BMI (Kg/M^2^), mean ± SD	22.6 ± 2.1	22.5 ± 1.9	22.8 ± 2.3	0.337
GCS, Mean ± SD	11.7 ± 1.5	11.8 ± 1.6	11.6 ± 1.5	0.345
NIHSS, Mean ± SD	11.7 ± 3.8	11.7 ± 4.0	11.9 ± 3.6	0.725
Site of hematoma, *n* (%)				0.696
Basal ganglia	67 (46.9)	47 (50)	20 (40.8)	
Supratentorial intracerebral	26 (18.2)	16 (17)	10 (20.4)	
Thalamic	29 (20.3)	17 (18.1)	12 (24.5)	
Infratentorial cerebellum	21 (14.7)	14 (14.9)	7 (14.3)	
Intraventricular hemorrhage, *n* (%)				0.447
No	102 (71.3)	69 (73.4)	33 (67.3)	
Yes	41 (28.7)	25 (26.6)	16 (32.7)	
CHV1 (ML, admission), mean ± SD	14.9 ± 6.8	15.3 ± 6.9	14.3 ± 6.8	0.408
CHV2 (ML, 3–5 days), mean ± SD	15.2 ± 6.6	15.5 ± 7.1	14.6 ± 6.6	0.325
PHE (ML, 3–5 Days), mean ± SD	22.3 ± 13.0	18.0 ± 8.7	30.6 ± 15.7	<0.001
Edema index, mean ± SD	1.5 ± 0.5	1.2 ± 0.2	2.1 ± 0.4	<0.001
Smoking (yes), *n* (%)	66 (46.2)	49 (52.1)	17 (34.7)	0.018
Alcohol consumption (yes), *n* (%)	57 (39.8)	45 (47.8)	12 (24.5)	0.011
History of hypertension (yes), *n* (%)	132 (92.3)	89 (94.7)	43 (87.8)	0.842
History of diabetes, *n* (%)	16 (11.2)	12 (12.8)	4 (8.2)	0.215
ANC (10^9^/L, admission), mean ± SD	4.8 ± 1.4	4.6 ± 1.3	5.1 ± 1.4	0.014
ALC (10^9^/L, admission), mean ± SD	1.3 ± 0.4	1.4 ± 0.4	1.3 ± 0.4	0.04
NLR1 (admission), mean ± SD	3.8 ± 1.2	3.5 ± 1.0	4.3 ± 1.2	<0.001
ANC (10^9^/L, 3 days), mean ± SD	6.0 ± 1.3	6.0 ± 1.3	5.9 ± 1.4	0.847
ALC (10^9^/L, 3 days), mean ± SD	1.3 ± 0.4	1.3 ± 0.4	1.3 ± 0.4	0.285
NLR2 (3–5 days), mean ± SD	5.8 ± 2.5	5.6 ± 2.5	6.0 ± 2.6	0.346

BMI, body mass index; GCS, Glasgow Coma Scale; NIHSS, National Institutes of Health Stroke Scale; CHV, cerebral hematoma volume; PHE, perihematoma edema; ANC, absolute neutrophil count; ALC, absolute lymphocyte count; NLR, neutrophil lymphocyte ratio.

### Assessment of prime results

The primary endpoint of this study was to extract data on perihematoma oedema in patients with conservatively treated intracerebral hemorrhage at the peak of their oedema. In this study, the open source software 3D Slicer was used to measure the volume of haematoma and oedema. Oedema index = volume of perihematoma oedema/volume of haematoma. In this study, 94 patients with an oedema index of 1.2 ± 0.2 and their perihematoma oedema of 18.0 ± 7.7 ML were included in the mild group. While 49 patients with an oedema index of 2.1 ± 0.4 and their perihematoma oedema of 30.6 ± 9.7 ML were included in the moderate to severe group (all *p* < 0.001, Table 1).

### Curve fitting analysis

**Figure 3:**
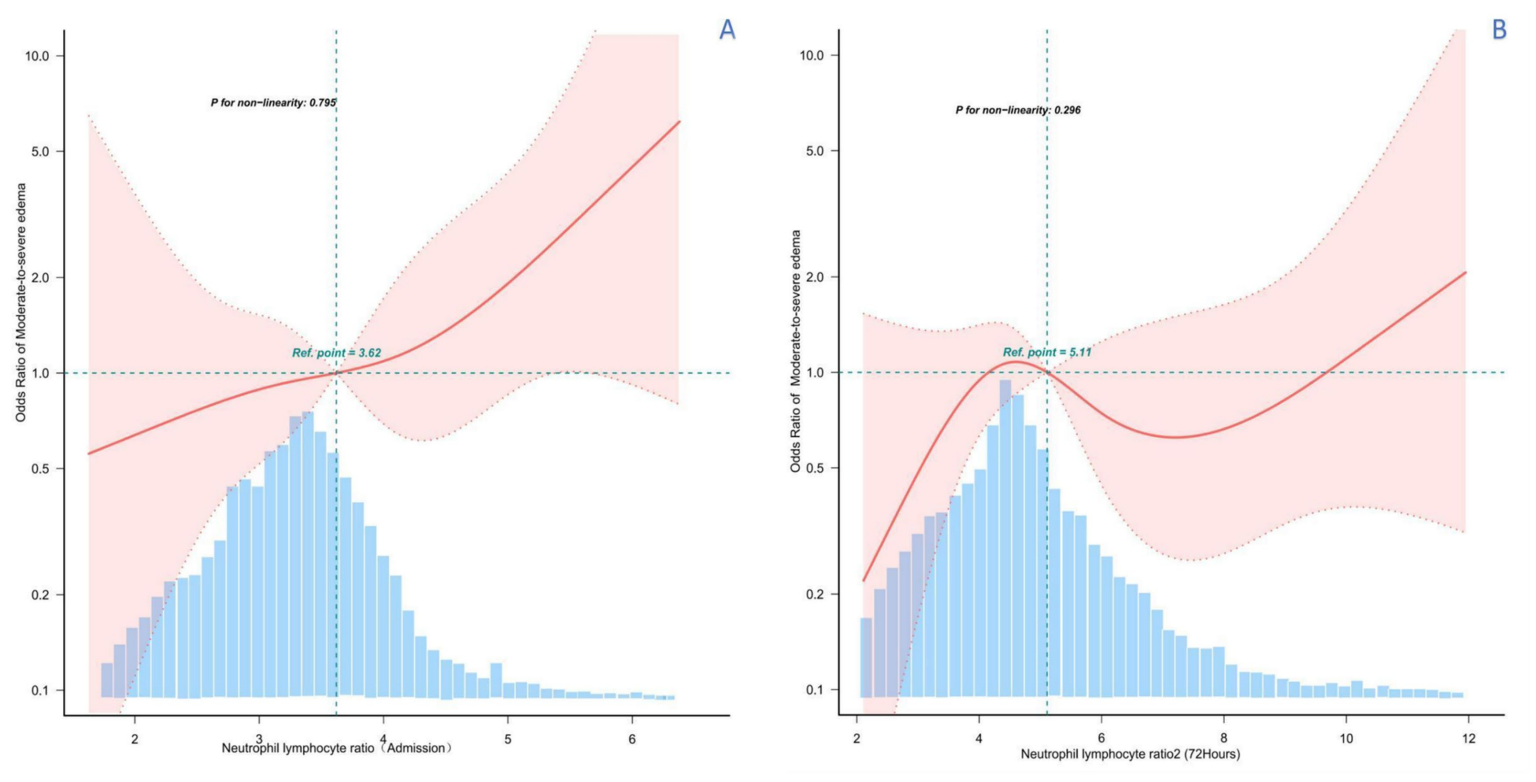
Restricted cubic spline analysis of the potential relationship between NLR and PHE at different time periods. **(A)** RCS analysis between NLR levels at admission and PHE, suggesting a potential linear positive relationship. **(B)** RCS analysis between NLR levels at 3–5 days of admission and treatment and PHE, showing an irregular relationship.

Smoothed curves were constructed using a restricted cubic spline model representing the risk relationship between exposure and PHE with NLR as the exposure. The solid red line represents the smoothed curve fit between the variables and the shaded bands indicate 95% confidence intervals. As shown in Figure 3, there was a potentially linear positive association between NLR1 on admission and PHE (*P* for non-linearity = 0.795). In contrast, there was no specific linear or curvilinear relationship between NLR2 and PHE during treatment.

### Univariate logistic regression analysis

In this study, univariate logistic regression analysis was used to investigate the independent effects of various variables, including NLR, on PHE. The results showed statistically significant differences between absolute neutrophil count (admission) (OR = 1.38, 95% CI: 1.06 ~ 1.8, *p* = 0.018), absolute lymphocyte count (admission) (OR = 0. 34, 95% CI: 0.12 ~ 0.97, *p* = 0.043), neutrophil-lymphocyte ratio1 (OR = 1.85, 95% CI: 1.33 ~ 2.59, *p* < 0.001) and smoking (OR = 2.34, 95% CI: 1.14 ~ 4.82, *p* = 0.021) and moderate to severe PHE (Table 2).

**Table 2:** Univariate analysis affecting the edema index.

Variable	OR_95 CI	*P*_value
Age, years	0.99 (0.95 ~ 1.03)	0.486
Gender, female	0.53 (0.26 ~ 1.07)	0.077
BMI	1.09 (0.92 ~ 1.28)	0.336
GCS	0.9 (0.71 ~ 1.12)	0.342
NIHSS	1.02 (0.93 ~ 1.11)	0.723
Site of hematoma, basal ganglia		
Supratentorial intracerebral	1.47 (0.57 ~ 3.79)	0.427
Thalamic	1.66 (0.67 ~ 4.1)	0.273
Infratentorial cerebellum	1.17 (0.41 ~ 3.35)	0.763
Intraventricular hemorrhage	1.34 (0.63 ~ 2.84)	0.448
Cerebral hematoma volume	0.98 (0.93 ~ 1.03)	0.405
Absolute neutrophil count (admission)	1.38 (1.06 ~ 1.8)	0.018
Absolute lymphocyte count (admission)	0.34 (0.12 ~ 0.97)	0.043
Neutrophil lymphocyte ratio 1	1.85 (1.33 ~ 2.59)	<0.001
Absolute neutrophil count (72 hours)	0.97 (0.75 ~ 1.27)	0.846
Absolute lymphocyte count (72 hours)	0.6 (0.23 ~ 1.54)	0.284
Neutrophil lymphocyte ratio 2	1.07 (0.93 ~ 1.22)	0.344
Smoking, yes	2.34 (1.14 ~ 4.82)	0.021
Alcohol consumption, yes	0.73 (0.36 ~ 1.47)	0.375

BMI, body mass index; GCS, Glasgow Coma Scale; NIHSS, National Institutes of Health Stroke Scale.

### Multi-variable logistic regression analysis

The study then continued with Logistic regression analysis to construct four models to examine the effect of NLR on PHE at different time points, adjusting for different covariates. The derived effect sizes (ORs) and their corresponding 95% confidence intervals are shown in Table 3. It was observed that the first NLR on admission was stably associated with PHE in all models, both when used as a continuous and categorical variable. In contrast, there was no statistically significant difference between NLR2 and PHE during hospitalization. Specifically, in the original model, when NLR1 was used as a continuous variable, there was a significant positive association with the risk of developing moderate to severe PHE (OR = 1.85, 95% CI: 1.33 to 2.59, *p* < 0.001). Adjustment for age and sex in model 2 showed stable results (OR = 1.79, 95% CI: 1.28 to 2.52, *p* = 0.001). In model 3, we continued to adjust for site of haematoma, intraventricular hemorrhage, GCS and NIHSS and the results remained stable (OR = 1.79, 95% CI: 1.19 ~ 2.68, *p* = 0.005). In model 4, we further adjusted for body mass index, hypertension, diabetes, and alcohol consumption, and alcohol consumption and the results remained stable (OR = 1.61, 95% CI: 1.03 ~ 2.5, *p* = 0.035).

**Table 3:** Logistic multivariate regression analysis model to assess the relationship between NLR and the risk of moderate-to-severe peripheral edema.

Variable	Model 1		Model 2		Model 3		Model 4	
	Crude OR (95CI)	*P*-value	Adjusted OR (95CI)	*P*-value	Adjusted OR (95CI)	*P*-value	Adjusted OR (95CI)	*P*-value
NLR 1 (admission, 1 SD)	1.85 (1.33 ~ 2.59)	< 0.001	1.79 (1.28 ~ −2.52)	0.001	1.79 (1.19 ~ −2.68)	0.005	1.61(1.03 ~ 2.5)	0.035
Quartile 1 (≤3.62)	1(Ref)		1(Ref)		1(Ref)		1(Ref)	
Quartile 2 (>3.62)	2.91 (1.14 ~ −6.0)	0.004	2.66 (1.26 ~ −5.62)	0.012	2.53(1.47 ~ −4.76)	0.027	2.47 (1.59 ~ −3.92)	0.021
NLR 2 (3–5 Days, 1 SD)	1.07 (0.93 ~ 1.22)	0.344	1.07 (0.93 ~ 1.23)	0.316	1.07 (0.95 ~ 1.44)	0.133	1.07 (0.95 ~ 1.45)	0.138
Quartile 1 (≤5.11)	1(Ref)		1(Ref)		1(Ref)		1(Ref)	
Quartile 2 (>5.11)	1.18 (0.59 ~ 2.36)	0.64	1.32 (0.65 ~ 2.68)	0.449	1.58 (0.6 ~ 4.17)	0.351	1.23 (0.45 ~ 3.35)	0.679

GCS, Glasgow Coma Scale; NIHSS, National Institutes of Health Stroke Scale.

Model 1: unadjusted; Model 2: adjusted for age and gender; Model 3: adjusted for Model 2 + (Site of hematoma, Intraventricular hemorrhage, GCS, NIHSS); Model 4: adjusted for Model3 + (Body mass index, Hypertension, Diabetes, Cigarette smoking and Alcohol drinking).

The groups were divided into higher and lower groups according to the median NLR. Covariate-adjusted model analyses were again performed separately. In the unadjusted model, the higher NLR group had a higher risk of moderate PHE (OR = 2.91, 95% CI: 1.14–6.0, *p* = 0.004). In the model adjusted for all covariates, the higher NLR group still had a higher risk of moderate to severe PHE (OR = 2.47, 95% CI: 1.59 ~ 3.92, *p* = 0.021). No statistically significant differences were found when NLR2 was analyzed as either a continuous or categorical variable in the model (Table 3).

### Receiver operating characteristic analysis

Based on ROC curve analysis, NLR may showed significant diagnostic value in predicting perihematoma oedema. In this study, the AUC value of NLR 1 was 0.719 (95% CI: 0.685–0.772), indicating its high sensitivity and specificity. In contrast, NLR 2 had an AUC value of 0.546 (95% CI: 0.447–0.645) and a *p*-value of 0.02, indicating that the diagnostic performance of NLR 1 was significantly better than that of NLR 2 (Supplementary Figure 1). These results are consistent with previous studies showing that NLR is a validated biomarker for assessing the prognosis of patients with cerebral hemorrhage. Therefore, NLR on admission may be a useful tool in the assessment of perihematoma oedema, contributing to clinical decision making and patient management.

### Subgroup analysis

**Figure 4:**
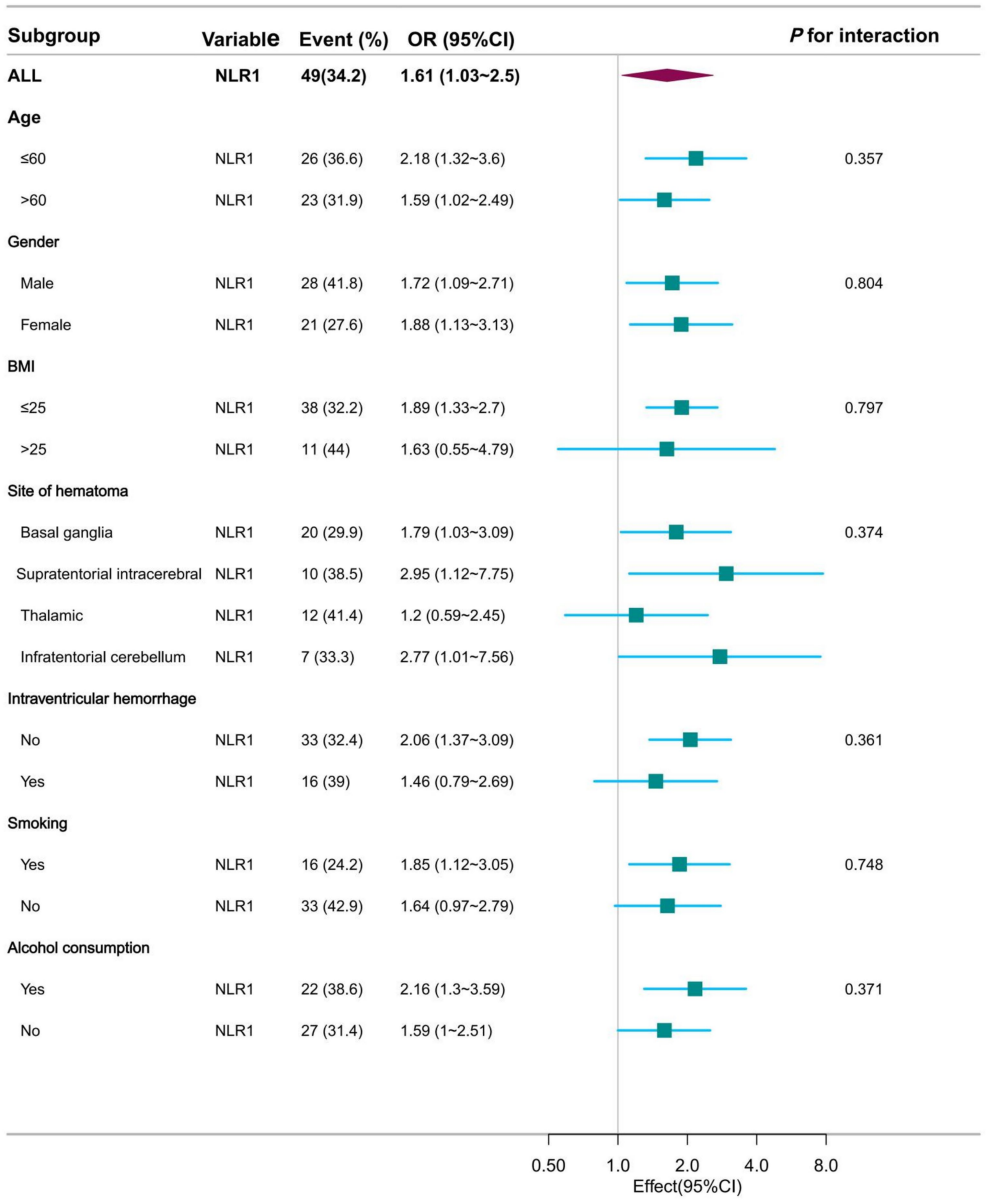
Forest plot presentation of subgroup analysis results when NLR was used as a continuous variable at admission.

**Figure 5:**
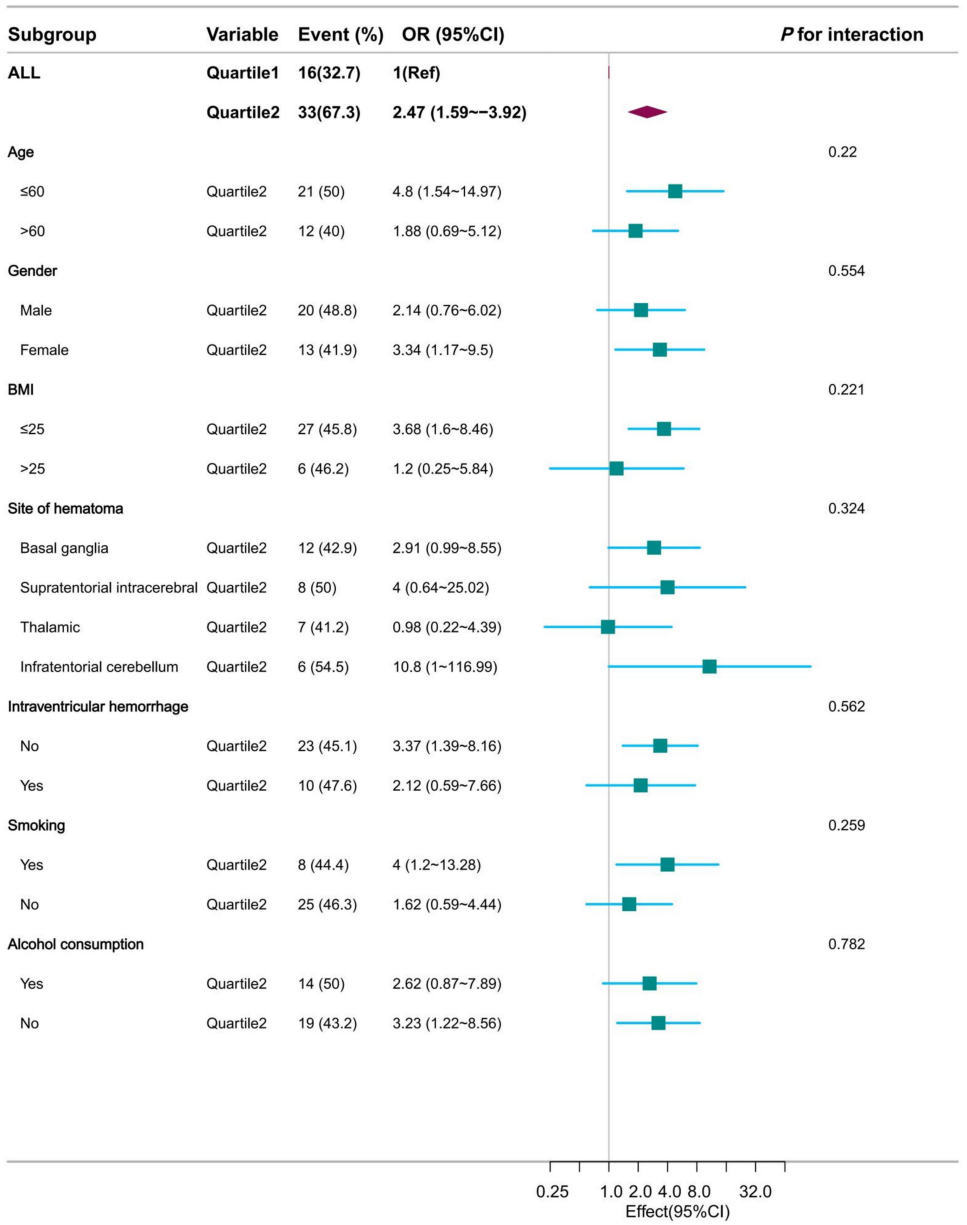
Forest plot presentation of subgroup analysis results when NLR was used as a categorical variable at admission.

This study was then analyzed in subgroups according to age, sex, BMI, haematoma location, intraventricular hemorrhage, smoking and alcohol consumption. Their subgroup results showed that this association remained stable in all subgroups, whether NLR1 was used as a continuous or categorical variable, and was consistent with the overall findings. Specifically, when NLR1 was used as a continuous variable, the associations between NLR1 and moderate to severe perihematoma oedema were 2.18 (95% CI: 1.32–3.6) and 1.59 (95% CI: 1.02–2.49) in patients aged ≤60 and >60 years, respectively, both of which were statistically significantly different. Similar associations were observed in other subgroups (Figure 4 and Supplementary Table 1). When NLR1 was used as a categorical variable (NLR > 3.62), ORs for all subgroups showed a positive association between the high NLR group and moderate to severe perihematomal oedema. For example, in patients with a BMI ≤ 25, the OR was 3.68 (95% CI: 1.6 to 8.46), whereas in patients with a BMI > 25, the trend was similar despite the smaller sample size (Figure 5 and Supplementary Table 2).

## Discussion

The aim of this study was to evaluate the relationship between PHE and NLR in patients with hypertensive cerebral hemorrhage. Considering NLR as a dynamically changing variable, this study was designed to analyze data from two time periods separately. The results of this study showed a significant positive linear correlation between admission NLR as a continuous variable and PHE in patients with moderate volume hypertensive cerebral hemorrhage. In a subsequent analysis of categorical variables, the higher NLR subgroup was shown to have a higher risk of moderate to severe PHE than the lower NLR group. This finding was consistent and significantly different in subsequent subgroup analyses. In contrast, there was no statistically significant difference between the NLR value at approximately 3–5 days during hospitalization and the study outcome. Finally, we further confirmed the higher sensitivity and specificity of NLR1 at admission than NLR2 during treatment with statistically significant differences using ROC curve analysis. Combined with these findings, NLR at admission has potential clinical application in predicting PHE in hypertensive cerebral hemorrhage.

The inflammatory response and haematoma expansion following cerebral hemorrhage play a critical role in predicting neurological impairment and disease prognosis (9). In the early post-hemorrhage phase, an inflammatory cascade is initiated leading to activation of microglia and astrocytes and infiltration of neutrophils and lymphocytes into the brain tissue (19). Neutrophils are usually the first responders to inflammation and cause tissue damage by releasing reactive oxygen species and protein hydrolases (20). On the other hand, lymphocytes play a role in regulating the immune response and promoting tissue repair (21), and NLR, as a measure of this balance, is increasingly recognized as a prognostic indicator for several neurological disorders, including stroke (22, 23). In our study, we observed a significant positive correlation between NLR and PHE on admission, which is consistent with previous findings that the higher the NLR, the worse the prognosis of stroke patients (24). Our findings extend this knowledge by demonstrating a specific correlation between NLR and PHE, which is an important predictor of neurological deterioration and poor prognosis after intracerebral hemorrhage. The ability to predict PHE early in the clinical course may help to guide targeted therapies to reduce oedema and improve patient prognosis (9).

Considering the temporal dynamics of NLR, this study defined NLR on admission as NLR1 and NLR after approximately 72 h of hospitalization as NLR2. The potential relationship between NLR and PHE at different time points was examined separately. After detailed analyses of NLR1 as a continuous and categorical variable respectively, the results further emphasized the potential relationship between NLR and PHE with statistically significant differences. The subgroup of patients with higher NLRs had a higher risk of developing moderate to severe PHE, reinforcing the importance of NLR in the prognosis of hypertensive cerebral hemorrhage. This finding was consistent across all subgroups analyzed, suggesting that the potential association of NLR 1 is robust and not influenced by other patient characteristics. In contrast, there was no statistically significant difference in NLR levels after 72 h of hospitalization, an interesting finding. This may suggest that the initial inflammatory response, as reflected by the NLR on admission, is more predictive of the subsequent development of PHE than the ongoing inflammatory state in the intervening period (25). Furthermore, the NLR during hospitalization is subject to greater bias as the disease progresses and therapy intervenes. This is consistent with the notion that early immune responses are critical in determining the severity of brain injury and subsequent oedema formation (26). ROC curve analyses provide additional evidence of the clinical utility of the admission NLR, which has greater sensitivity and specificity than the treatment NLR. This further confirms the statistically significant difference between Admission NLR1 and Treatment NLR2 and highlights the importance of early assessment in predicting PHE (27).

### Other inflammatory markers and their relationship with NLR

Several other inflammatory markers have also been associated with the development of PHE. For instance, elevated levels of C-reactive protein (CRP) and interleukin-6 (IL-6) suggest an elevated likelihood of PHE in patients with cerebral hemorrhage. Like NLR, these markers reflect the systemic inflammatory response following brain injury. The interplay between these markers and NLR is complex. For instance, high levels of CRP and IL-6 may contribute to the disruption of the blood–brain barrier (BBB), which is a key factor in PHE development (23). As a ratio of neutrophils to lymphocytes, NLR provides a balanced view of the inflammatory response: neutrophils promote inflammation, while lymphocytes have a regulatory role. This balance may influence the overall inflammatory response and thus affect the severity of PHE.

### Potential pathophysiological mechanisms of NLR on PHE

The positive correlation between NLR and PHE suggests the existence of several such mechanisms. Neutrophils, which constitute the majority of NLR cells, release ROS and proteolytic enzymes that can damage the BBB and exacerbate cerebral oedema. Lymphocytes, on the other hand, play a role in modulating the immune response and promoting tissue repair (21). An elevated NLR indicates a predominance of neutrophils, suggesting a more pro-inflammatory state that could lead to increased PHE. Furthermore, the activation of microglia and astrocytes following cerebral hemorrhage can amplify the inflammatory response, thereby contributing to PHE development. Dynamic changes in NLR over time may reflect an evolving inflammatory response, with high initial neutrophil levels driving the early formation of PHE.

### Hypothesized pathways and suggestions for further research

Further research should explore the detailed mechanisms by which NLR influences PHE. One potential pathway involves the interaction between neutrophils and the BBB. Studies could examine the role of neutrophil extracellular traps (NETs) in disrupting the BBB and exacerbating PHE. Additionally, the impact of lymphocyte subsets, such as regulatory T cells, on the inflammatory response and PHE should be investigated (19). Future studies should also consider the temporal dynamics of NLR and other inflammatory markers, using longitudinal sampling to capture the evolution of the inflammatory response. This could provide insights into the optimal timing for interventions aimed at modulating inflammation and reducing PHE. Furthermore, clinical trials could evaluate the effectiveness of targeted therapies, such as anti-inflammatory agents or immunomodulatory drugs, in reducing NLR levels and improving patient outcomes in cases of hypertensive cerebral hemorrhage.

### Strengths and limitations

The present study has several strengths in its analysis of the relationship between NLR and PHE in patients with hypertensive cerebral hemorrhage. First, our multicentre retrospective design provides a diverse patient cohort, increasing the generalisability of our findings to diverse populations in southwest China. The use of restricted cubic spline and logistic regression modeling allows a nuanced examination of the association between NLR and PHE, capturing non-linear relationships that may be missed by simpler statistical approaches. In addition, longitudinal assessment of NLR at admission and during hospitalization provides insight into the dynamic nature of NLR as a potential biomarker. The large sample size and comprehensive data collection, including demographics, medical history and lesion characteristics, further strengthen the robustness of our findings, but the study is not without its limitations. The retrospective nature of the study limits our ability to establish causality. Although we have adjusted for several covariates in our analyses, we acknowledge that residual confounding factors may still be influencing the observed associations. It is not possible to rule out completely the possibility that unmeasured confounding factors could affect the relationship between NLR and PHE. For example, the timing and type of treatment interventions, genetic predispositions and other unrecorded comorbidities could affect the outcomes. Furthermore, while our ROC curve analysis suggests that NLR at admission has diagnostic value, the predictive value of NLR requires validation in prospective studies involving larger, more diverse cohorts. Therefore, future studies should aim to address these limitations by collecting more comprehensive data and following patients over time in order to better understand the temporal dynamics of NLR and its relationship with PHE. Finally, focusing the study on a specific population in southwestern China could limit the generalisability of our findings to other regions with different ethnic and socioeconomic profiles. For example, studies have demonstrated that the prevalence and severity of intracerebral hemorrhage can vary significantly between populations due to genetic, environmental and lifestyle factors (1). For example, Song et al. (2) found that the global burden of intracerebral hemorrhage was higher in regions with lower socioeconomic levels, potentially due to differences in healthcare access and lifestyle factors such as smoking and alcohol consumption. Similarly, genetic predispositions can influence the inflammatory response and the development of cerebral oedema, as demonstrated in studies on the role of genetic polymorphisms in stroke pathogenesis (2, 19). Therefore, our findings may not be directly applicable to populations with different genetic backgrounds or living in different socioeconomic conditions. Reliance on electronic medical records for data extraction introduces potential biases, including missing data and variability in data quality. Future studies should aim to include more diverse populations to better understand the generalisability of our findings.

## Conclusion

This study confirms the potential association between higher NLR on admission and increased risk of moderate to severe PHE in patients with hypertensive cerebral hemorrhage. It also confirms the role of systemic inflammation in secondary brain injury as supported by previous literature. NLR is a readily available biomarker and its potential association with PHE provides a valuable tool for early identification of high-risk patients. However, due to the inherent limitations of our retrospective design, future prospective studies are needed to confirm these findings and explore the underlying mechanisms, thereby increasing the clinical utility of NLR in the management of cerebral hemorrhage prognosis.

## Data Availability

The original contributions presented in the study are included in the article/Supplementary material, further inquiries can be directed to the corresponding author.
