# Supplementary material for: The association between neutrophil lymphocyte ratio and perihematomal edema in cerebral hemorrhage: a multicenter retrospective study
Source: Front Neurol. 2025 Jul 4;16:1575446. doi: 10.3389/fneur.2025.1575446 (PMC12270894; doi:10.3389/fneur.2025.1575446)
Supplement: Supplementary file 1 [file Table_1.docx]

**S Table1** Subgroup analysis of the relationship between NLR and peripheral edema in moderate-to-severe hematomas.

| **Subgroup** | **Variable** | **n.total** | **n.event_%** | **Crude.**  **OR_95CI** | **Crude.**  ***P*_value** | ***P*.for.interaction** |
| --- | --- | --- | --- | --- | --- | --- |
| Age |  |  |  |  |  |  |
| ≤60 | NLR1 | 71 | 26 (36.6) | 2.18 (1.32~3.6) | 0.002 | 0.357 |
| >60 | NLR1 | 72 | 23 (31.9) | 1.59 (1.02~2.49) | 0.042 |  |
| Gender |  |  |  |  |  |  |
| Male | NLR1 | 67 | 28 (41.8) | 1.72 (1.09~2.71) | 0.019 | 0.804 |
| Female | NLR1 | 76 | 21 (27.6) | 1.88 (1.13~3.13) | 0.015 |  |
| BMI |  |  |  |  |  |  |
| ≤25 | NLR1 | 118 | 38 (32.2) | 1.89 (1.33~2.7) | <0.001 | 0.797 |
| >25 | NLR1 | 25 | 11 (44) | 1.63 (0.55~4.79) | 0.374 |  |
| Site of hematoma |  |  |  |  |  |  |
| Basal ganglia | NLR1 | 67 | 20 (29.9) | 1.79 (1.03~3.09) | 0.038 | 0.374 |
| Supratentorial intracerebral | NLR1 | 26 | 10 (38.5) | 2.95 (1.12~7.75) | 0.028 |  |
| Thalamic | NLR1 | 29 | 12 (41.4) | 1.2 (0.59~2.45) | 0.61 |  |
| Infratentorial cerebellum | NLR1 | 21 | 7 (33.3) | 2.77 (1.01~7.56) | 0.047 |  |
| Intraventricular hemorrhage |  |  |  |  |  |  |
| No | NLR1 | 102 | 33 (32.4) | 2.06 (1.37~3.09) | <0.001 | 0.361 |
| Yes | NLR1 | 41 | 16 (39) | 1.46 (0.79~2.69) | 0.224 |  |
| Smoking |  |  |  |  |  |  |
| Yes | NLR1 | 66 | 16 (24.2) | 1.85 (1.12~3.05) | 0.016 | 0.748 |
| No | NLR1 | 77 | 33 (42.9) | 1.64 (0.97~2.79) | 0.066 |  |
| Alcohol consumption |  |  |  |  |  |  |
| Yes | NLR1 | 57 | 22 (38.6) | 2.16 (1.3~3.59) | 0.003 | 0.371 |
| No | NLR1 | 86 | 27 (31.4) | 1.59 (1~2.51) | 0.049 |  |

**Abbreviation:**BMI,Body mass index; NLR1,Neutrophil lymphocyte ratio(Admission). A stable positive correlation between NLR and moderate-to-severe perihematoma edema was found in all subgroups.
