# Supplementary material for: The association between neutrophil lymphocyte ratio and perihematomal edema in cerebral hemorrhage: a multicenter retrospective study
Source: Front Neurol. 2025 Jul 4;16:1575446. doi: 10.3389/fneur.2025.1575446 (PMC12270894; doi:10.3389/fneur.2025.1575446)
Supplement: Supplementary file 2 [file Table_2.docx]

**S Table2** Subgroup analysis of the relationship between NLR(categorical variable) and peripheral edema in moderate-to-severe hematomas.

| **Subgroup** | **Variable** | **N.total** | **N.even%** | **Crude.**  **OR_95CI** | **Crude.**  ***P*_value** | 1. **for.**   **interaction** |
| --- | --- | --- | --- | --- | --- | --- |
| Age |  |  |  |  |  | 0.22 |
| ≤60 | Quartile2 | 42 | 21 (50) | 4.8 (1.54~14.97) | 0.007 |  |
| >60 | Quartile2 | 30 | 12 (40) | 1.88 (0.69~5.12) | 0.218 |  |
| Gender |  |  |  |  |  | 0.554 |
| Male | Quartile2 | 41 | 20 (48.8) | 2.14 (0.76~6.02) | 0.148 |  |
| Female | Quartile2 | 31 | 13 (41.9) | 3.34 (1.17~9.5) | 0.024 |  |
| BMI |  |  |  |  |  | 0.221 |
| ≤25 | Quartile2 | 59 | 27 (45.8) | 3.68 (1.6~8.46) | 0.002 |  |
| >25 | Quartile2 | 13 | 6 (46.2) | 1.2 (0.25~5.84) | 0.821 |  |
| Site of hematoma |  |  |  |  |  | 0.324 |
| Basal ganglia | Quartile2 | 28 | 12 (42.9) | 2.91 (0.99~8.55) | 0.053 |  |
| Supratentorial intracerebral | Quartile2 | 16 | 8 (50) | 4 (0.64~25.02) | 0.138 |  |
| Thalamic | Quartile2 | 17 | 7 (41.2) | 0.98 (0.22~4.39) | 0.979 |  |
| Infratentorial cerebellum | Quartile2 | 11 | 6 (54.5) | 10.8 (1~116.99) | 0.05 |  |
| Intraventricular hemorrhage |  |  |  |  |  | 0.562 |
| No | Quartile2 | 51 | 23 (45.1) | 3.37 (1.39~8.16) | 0.007 |  |
| Yes | Quartile2 | 21 | 10 (47.6) | 2.12 (0.59~7.66) | 0.251 |  |
| Smoking |  |  |  |  |  | 0.259 |
| Yes | Quartile2 | 18 | 8 (44.4) | 4 (1.2~13.28) | 0.024 |  |
| No | Quartile2 | 54 | 25 (46.3) | 1.62 (0.59~4.44) | 0.352 |  |
| Alcohol consumption |  |  |  |  |  | 0.782 |
| Yes | Quartile2 | 28 | 14 (50) | 2.62 (0.87~7.89) | 0.086 |  |
| No | Quartile2 | 44 | 19 (43.2) | 3.23 (1.22~8.56) | 0.018 |  |

**Abbreviation:**BMI,Body mass index; NLR,Neutrophil lymphocyte ratio;Quartile2( NLR>3.62). When NLR was used as the categorical variable and Quartile1 was used as the reference, a stable positive correlation between high grouping (Quartile2) and moderate to severe perihematomal edema was still found in all subgroups.
