# Supplementary figures and images for: The association between neutrophil lymphocyte ratio and perihematomal edema in cerebral hemorrhage: a multicenter retrospective study
Source: Front Neurol. 2025 Jul 4;16:1575446. doi: 10.3389/fneur.2025.1575446 (PMC12270894; doi:10.3389/fneur.2025.1575446)

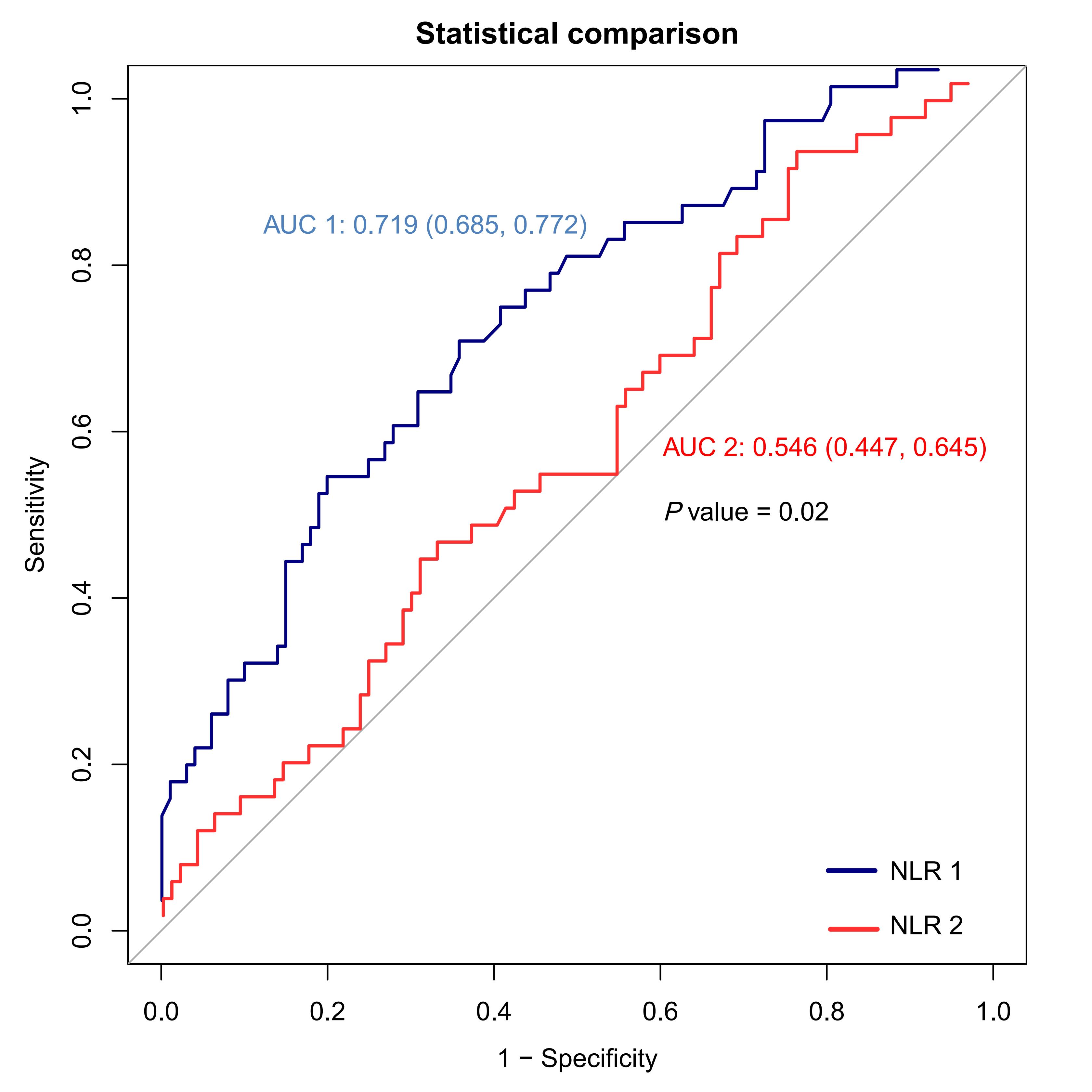

Supplement: Supplementary file 3 [file Image_1.jpeg]
